# Toward a refined classification of class I dithiol glutaredoxins from poplar: biochemical basis for the definition of two subclasses

**DOI:** 10.3389/fpls.2013.00518

**Published:** 2013-12-18

**Authors:** Jérémy Couturier, Jean-Pierre Jacquot, Nicolas Rouhier

**Affiliations:** ^1^Interactions Arbres - Microorganismes, Université de Lorraine, UMR1136Vandoeuvre-lès-Nancy, France; ^2^Interactions Arbres - Microorganismes, Institut National de la Recherche Agronomique, UMR1136Champenoux, France

**Keywords:** cysteine oxidation, deglutathionylation, disulfide bond, glutaredoxin, redox potential

## Abstract

Glutaredoxins (Grxs) are small oxidoreductases particularly specialized in the reduction of protein-glutathione adducts. Compared to other eukaryotic organisms, higher plants present an increased diversity of Grxs which are organized into four classes. This work presents a thorough comparative analysis of the biochemical and catalytic properties of dithiol class I Grxs from poplar, namely GrxC1, GrxC2, GrxC3, and GrxC4. By evaluating the *in vitro* oxidoreductase activity of wild type and cysteine mutated variants and by determining their dithiol-disulfide redox potentials, p*K*_*a*_ values of the catalytic cysteine, redox state changes in response to oxidative treatments, two subgroups can be distinguished. In accordance with their probable quite recent duplication, GrxC1 and GrxC2 are less efficient catalysts for the reduction of dehydroascorbate and hydroxyethyldisulfide compared to GrxC3 and GrxC4, and they can form covalent dimers owing to the presence of an additional C-terminal cysteine (Cys_*C*_). Interestingly, the second active site cysteine (Cys_B_) influences the reactivity of the catalytic cysteine (Cys_A_) in GrxC1 and GrxC2 as already observed with GrxC5 (restricted to *A. thaliana*), but not in GrxC3 and C4. However, all proteins can form an intramolecular disulfide between the two active site cysteines (Cys_A_-Cys_B_) which could represent either a protective mechanism considering that this second cysteine is dispensable for deglutathionylation reaction or a true catalytic intermediate occurring during the reduction of particular disulfide substrates or in specific conditions or compartments where glutathione levels are insufficient to support Grx regeneration. Overall, in addition to their different sub-cellular localization and expression pattern, the duplication and maintenance along evolution of several class I Grxs in higher plants can be explained by the existence of differential biochemical and catalytic properties.

## Introduction

Glutaredoxins (Grxs) are oxidoreductases sharing a conserved 3D structure with members of the thioredoxin (Trx) superfamily. They are present in most living organisms except in some bacterial and archaeal phyla (Rouhier et al., 2008; Alves et al., 2009). The primary function of Grxs was long thought to be the reduction of disulfide bonds and more particularly those formed between reduced glutathione (GSH) and a protein cysteinyl residue, a process known as glutathionylation, but as explained below, specific Grx members could rather serve as iron-sulfur (Fe-S) cluster transfer proteins (Rouhier, 2010). Glutathionylation is a post-translational modification which potentially fulfills several functions. It can constitute an intermediate of some reaction mechanisms, but it can also modulate protein function and serve as a signaling mechanism or protect cysteine residues from irreversible oxidation in plants (Zaffagnini et al., 2012b) and in animals (Dalle-Donne et al., 2009). Although a few additional proteins, as some specific thioredoxins, can catalyze deglutathionylation reactions, Grxs are likely the major deglutathionylation system, at least in plants (Bedhomme et al., 2012; Chibani et al., 2012). They are usually regenerated via a NADPH/glutathione reductase (GR)/GSH system, but a few Grxs can be reduced by ferredoxin- or NADPH-dependent thioredoxin reductases (Johansson et al., 2004; Zaffagnini et al., 2008). Depending on the Grx isoforms and target proteins or substrates considered, Grxs can use different catalytic mechanisms, called monothiol and dithiol mechanisms (Rouhier et al., 2008). The monothiol mechanism is used for the reduction of glutathionylated proteins and requires *a priori* only the catalytic cysteine (the first or more N-terminal of the two active site cysteines, referred to as Cys_A_). It performs a nucleophilic attack on the protein-glutathione adduct, the Grx becoming glutathionylated. This oxidized Grx is regenerated by reduction via a GSH molecule. The dithiol mechanism requires the catalytic cysteine but also a recycling cysteine which could be either the second active site cysteine (Cys_B_) or an additional extra active site cysteine (Cys_*C*_). If the target protein is glutathionylated, the first step is similar to the monothiol mechanism, but the glutathione-adduct formed on Grx is solved by one of these recycling cysteines instead of GSH. The resulting disulfide is then either reduced by two molecules of GSH or by a thioredoxin reductase. If the target protein has an intra or inter-molecular disulfide, the mechanism is comparable to the one used by Trxs. The catalytic cysteine forms a transient mixed disulfide with one of the cysteines of the target protein, which is then resolved by the recycling cysteine. As above, final regeneration occurs via two molecules of GSH or via a thioredoxin reductase.

Based on the active site sequence, Grxs were initially categorized into two classes, a dithiol (CP[Y/F]C motif) and a monothiol (CGFS motif) class (Rodriguez-Manzaneque et al., 1999) that were subsequently renamed classes I and II, respectively (Couturier et al., 2009a). Analyses of Grx distribution and evolution in archaea, bacteria, and eukaryotes suggested that Grx domains of classes I and II have evolved through duplication and divergence from one initial gene present in the last common ancestor of all organisms (Alves et al., 2009). Two additional classes are found in land plants (Couturier et al., 2009a). The Grx isoforms belonging to class III are specific to land plants and those from class IV are also present in few algae and animals. There is no biochemical or functional information on the latter Grxs yet. From genetic analyses, it appears that class III Grx isoforms also named CC-type Grxs or ROXY play a role in plant development and in pathogen defense mechanisms, reviewed in (Meyer et al., 2012). These functions are related to their ability to interact with TGA transcription factors, likely regulating their redox states. However, the biochemical properties of these Grxs have not been studied in detail yet, because of the difficulty to produce soluble recombinant proteins (Couturier et al., 2010). Only one study demonstrated that *Arabidopsis thaliana* GrxS10 is able to regenerate *in vitro* the mitochondrial type IIF peroxiredoxin (Finkemeier et al., 2005). The Grxs from class II are particular considering their involvement in the maturation of Fe-S clusters and in the regulation of iron homeostasis (Rouhier et al., 2010), which is likely related to their capacity to bind labile Fe-S clusters and to transfer them to target proteins (Bandyopadhyay et al., 2008). On the other hand, though many studies performed with class II Grxs from various sources indicated that they have no or very poor efficiency for the reduction of the traditionally tested glutathionylated substrates using a GR/GSH regeneration system, at least one study indicated that they have a reductase activity. Indeed, *Chlamydomonas reinhardtii* Grx3 is able to reduce *in vitro* a glutathionylated A_4_-glyceraldehyde-3-phosphate dehydrogenase (Zaffagnini et al., 2008). Interestingly, it can be regenerated by the ferredoxin-thioredoxin reductase but not by the GSH/GR system. The existence of alternative regeneration systems may explain why the characterization of such activities for class II Grxs has been retarded. Overall, the demonstrated capacity of several class I Grxs to provide electrons to a battery of enzymes, as peroxiredoxins and methionine sulfoxide reductases, enzymes that go through a catalytic cycle which involves glutathionylation of their catalytic cysteine (Rouhier et al., 2001; Vieira Dos Santos et al., 2007; Tarrago et al., 2009), suggests that they are the central and favorite deglutathionylating agents in cells.

In plants, class I regroups six different glutaredoxin members called GrxC1, C2, C3, C4, S12, and C5, the latter being restricted to Brassicaceae (Couturier et al., 2011). The cytoplasmic GrxC1 and the plastidial GrxC5 are able to bind an [Fe_2_S_2_] cluster, at least *in vitro* into recombinant proteins (Rouhier et al., 2007; Couturier et al., 2011). The biochemical and structural characterization of the two plastidial isoforms, GrxS12 and GrxC5, confirmed that the apoforms of these Grxs reduce glutathionylated proteins with catalytic properties quite similar to other plant and non-plant class I Grxs (Couturier et al., 2009b, 2011). Moreover, poplar GrxS12 was shown to be sensitive and temporarily inactivated after treatments with oxidizing compounds such as hydrogen peroxide (H_2_O_2_), nitrosoglutathione (GSNO), and oxidized glutathione (GSSG) (Zaffagnini et al., 2012a). Considering the very electronegative redox potential of the glutathione adduct formed on the catalytic Cys_A_ (−315 mV at pH 7.0), the reduction of GrxS12 necessitates a quite low redox potential of the GSH/GSSG couple in the chloroplast. Thus, GrxS12 may act as a stress-related redox regulator allowing glutathione to play a signaling role in some oxidizing conditions by maintaining the glutathionylation of its target proteins over the duration of the stress period. Several class I Grxs, *C. reinhardtii* Grx1 and Grx2 (Gao et al., 2010), *A. thaliana* GrxC1, C2 (Riondet et al., 2012) and GrxC5 (Couturier et al., 2011) and poplar GrxC1, GrxC4, and GrxS12 (Rouhier et al., 2002, 2007; Couturier et al., 2009b; Zaffagnini et al., 2012a) have been partially or extensively characterized at the biochemical level, but some members have not yet been studied and there is no thorough and comparative study allowing to understand if the existence of several members is only related to their sub-cellular localization or to their expression pattern or whether some biochemical properties can partially explain their diversity.

From the biochemical analyses performed here with the four dithiol class I Grxs existing in poplar, GrxC1 to GrxC4 (the fifth member, GrxS12, has a monothiol active site and was characterized previously), two sub-groups with distinct properties emerged. In addition, this work provides new insights on the existence of different oxidation forms involving the active site cysteines (Cys_A_ and Cys_B_) or an additional C-terminal cysteine (Cys_*C*_) and on their potential role for the reaction mechanism.

## Materials and methods

### Site-directed mutagenesis

The construction of pET3d expression plasmids containing poplar GrxC1, C2, C3, and C4 was described previously (Rouhier et al., 2002, 2007). All cysteine residues of GrxC2, C3 were individually substituted into serines using two complementary mutagenic primers (listed in Table S1). The corresponding mutated proteins are called GrxC2 C23S, C26S, or C80S, GrxC3 C37S or C40S. The recombinant proteins GrxC1 C31S, C34S, C88S, and GrxC4 C27S and C30S were prepared previously (Rouhier et al., 2002, 2007). Two additional mutants, in which only the catalytic cysteine is remaining, GrxC1 C34/88S and GrxC2 C26/80S, have also been cloned.

### Heterologous expression and purification of recombinant proteins in *E. coli*

For protein expression, cultures of the *E. coli* BL21(DE3) strain, containing the pSBET plasmid and co-transformed with the different recombinant pET3d plasmids, were successively amplified up to 2.4 L in LB medium at 37°C supplemented with 50 μg/mL of ampicillin and kanamycin. Induction of protein expression was performed at exponential phase by adding 100 μM isopropyl β-D-thiogalactopyranoside for 4 h at 37°C. After centrifugation (20 min at 4400 g), the cell pellets were resuspended in about 20 mL of TE NaCl buffer (30 mM Tris-HCl pH 8.0, 1 mM EDTA, 200 mM NaCl) and eventually conserved at −20°C. Cell lysis was performed by sonication and the soluble and insoluble fractions were separated by centrifugation for 45 min at 27,000 g.

Purification of proteins was carried out in three steps. The soluble fraction was first precipitated by ammonium sulfate from 0 to 40% and then to 80% of the saturation. The 40–80% ammonium sulfate-precipitated fraction was subjected to gel filtration chromatography (ACA44 gel) equilibrated with TE NaCl buffer. After dialysis against TE (30 mM Tris-HCl pH 8.0, 1 mM EDTA) buffer and concentration, the interesting fractions were loaded to a DEAE (diethylaminoethyl) sepharose column equilibrated in TE buffer. GrxC1 WT, GrxC2 WT and their corresponding cysteinic mutants passed through the column, whereas GrxC3 WT, GrxC4 WT and their respective cysteinic mutants were retained and eluted using a linear 0–0.4 M NaCl gradient. The purest fractions, as judged by SDS-PAGE analysis, were pooled and dialyzed against TE buffer by ultrafiltration in Amicon cells equipped with a YM10 membrane. Finally, the fractions were concentrated and stored at −20°C until further use. Protein purity was checked by SDS-PAGE, and protein concentrations were determined spectrophotometrically using the corresponding molar extinction coefficients at 280 nm of 10,095 M^−1^ cm^−1^ for GrxC1 WT and its monocysteinic mutants, 9970 M^−1^ cm^−1^ for GrxC1 C34/88S, 8605 M^−1^ cm^−1^ for GrxC2 WT and its monocysteinic mutants, 8480 M^−1^ cm^−1^ for GrxC2 C26/80S, 7575 M^−1^ cm^−1^ for GrxC3 WT, 7450 M^−1^ cm^−1^ for GrxC3 C37S and C40S, 4595 M^−1^ cm^−1^ for GrxC4 WT and 4470 M^−1^ cm^−1^ for GrxC4 C27S and C30S.

### Enzymatic activities

The thioltransferase activities [2-hydroxyethyl-disulfide (HED) and dehydroascorbate (DHA) assays] were measured as described previously (Couturier et al., 2011) using as purified non-reduced proteins. Briefly, measurements were performed at 25°C in steady-state conditions by following NADPH oxidation at 340 nm in the presence of a Grx reducing system which is composed of NADPH, GR, and GSH. The reaction was started by adding Grx after a 3-min pre-incubation time and Grx activity was corrected by subtracting the spontaneous reduction rate observed in the absence of Grx. Because of the spontaneous reaction of GSH with the substrates, we can generally not work at saturating concentrations of all substrates using these assays. Enzyme and substrate concentrations used are indicated in the legend of figures and tables. The activity was expressed as nmol of NADPH oxidized/nmol of Grx/s using a molar extinction coefficient of 6220 M^−1^ cm^−1^ at 340 nm for NADPH. Three independent experiments were performed at each substrate concentration, and *k*_*cat*_ and apparent *K*_*m*_ values were calculated by non-linear regression using the Michaelis–Menten equation using the program GraphPad Prism 4.

### Preparation and analysis of oxidized Grxs

Around 10 mg of protein was reduced using 40 mM DTT in 500 μl of 30 mM Tris-HCl pH 8.0 buffer for 2 h at 25°C followed by desalting on G25 column pre-equilibrated with 30 mM Tris-HCl pH 8.0 buffer. Oxidized proteins were prepared by incubating 500 μM reduced poplar Grxs in *ca* 200 μL with a 10 fold excess GSSG or H_2_O_2_ or a 5 fold excess GSNO for 2 h at 25°C before desalting on G25 columns. The protein oxidation state was then analyzed by electrospray ionization mass spectrometry analysis as described previously (Couturier et al., 2011).

For alkylation assays, reduced Grxs treated or not with oxidants were diluted to 10 μM into 50 μ l of 30 mM Tris-HCl pH 8.0 buffer and precipitated on ice for 30 min with one volume of 20% trichloroacetic acid (TCA). After centrifugation (10 min at 13,000 *g*) and washing with 100% acetone, the pellet was resuspended into 10 μl of 100 mM Tris-HCl pH 8.0, 1% SDS containing 2 mM of methoxyl-PEG maleimide of 2 kDa (mPEG maleimide) which alkylates free thiol groups. The protein mixture was then separated on non-reducing 15% SDS-PAGE.

For midpoint redox potential titrations, Cys_A_ glutathionylated forms were obtained by the above described GSNO treatment of the double cysteinic mutants (GrxC1 C34/88S, GrxC2 C26/80S) and of the monocysteinic mutants (GrxC3 C40S and GrxC4 C30S). The intramolecular disulfide bond (Cys_A_-Cys_B_) containing proteins were obtained by the above described H_2_O_2_ treatment but using GrxC1 C88S, GrxC2 C80S and GrxC3 and GrxC4 WT isoforms.

### P*K*_*a*_ determination of the catalytic cysteine

The p*K*_*a*_ measurements of N-terminal active site cysteines (Cys_A_) have been performed with WT Grx isoforms following a procedure described in (Gallogly et al., 2008). Briefly, 3 μM reduced Grx was incubated with or without 300 μM iodoacetamide (IAM) in 100 mM sodium citrate or phosphate buffers ranging from pH 2.0 to 7.0. Following this pre-incubation step, Grx activity was determined by adding an aliquot of the pre-incubation mixture to the HED assay described above. The percentages of remaining activity at each pH were determined by comparing the activity of the enzyme incubated with and without IAM and an adaptation of the Henderson–Hasselbach equation (Gallogly et al., 2008) was used for p*K*_*a*_ value determination using the program GraphPad Prism 4.

### Midpoint redox potential (*E*_*m*_) determination

Oxidation-reduction titrations using the fluorescence of the adduct formed between protein free thiols and monobromobimane (mBBr) were carried out at ambient temperature in 200 μl of 100 mM HEPES pH 7.0 buffer containing 10 μM of oxidized proteins, either glutathionylated or with an intramolecular disulfide-bond, and defined mixtures of oxidized and reduced DTT or glutathione for more positive values to set the ambient potential (*E*_*h*_). Total concentration of DTT or glutathione was 2 mM. After 2 h incubation, mBBr was added at a final concentration of 2 mM and the reaction was carried out in the dark for 20 min. The reaction mixture was then precipitated on ice for 30 min with one volume of 20% TCA. After centrifugation (10 min at 13,000 g) and washing with 100% acetone, the pellet was resuspended into 400 μl of 100 mM Tris-HCl pH 8.0, 1% SDS. Fluorescence emission of the resulting solution was measured at 472 nm after excitation at 380 nm using a variant Cary Eclipse (Agilent). Values were transformed into percentages of reduced protein and fitted to the Nernst equation using non-linear regression for *E*_*m*_ value determination.

## Results

### Kinetic comparison of poplar class I Grxs

**Figure 1:**
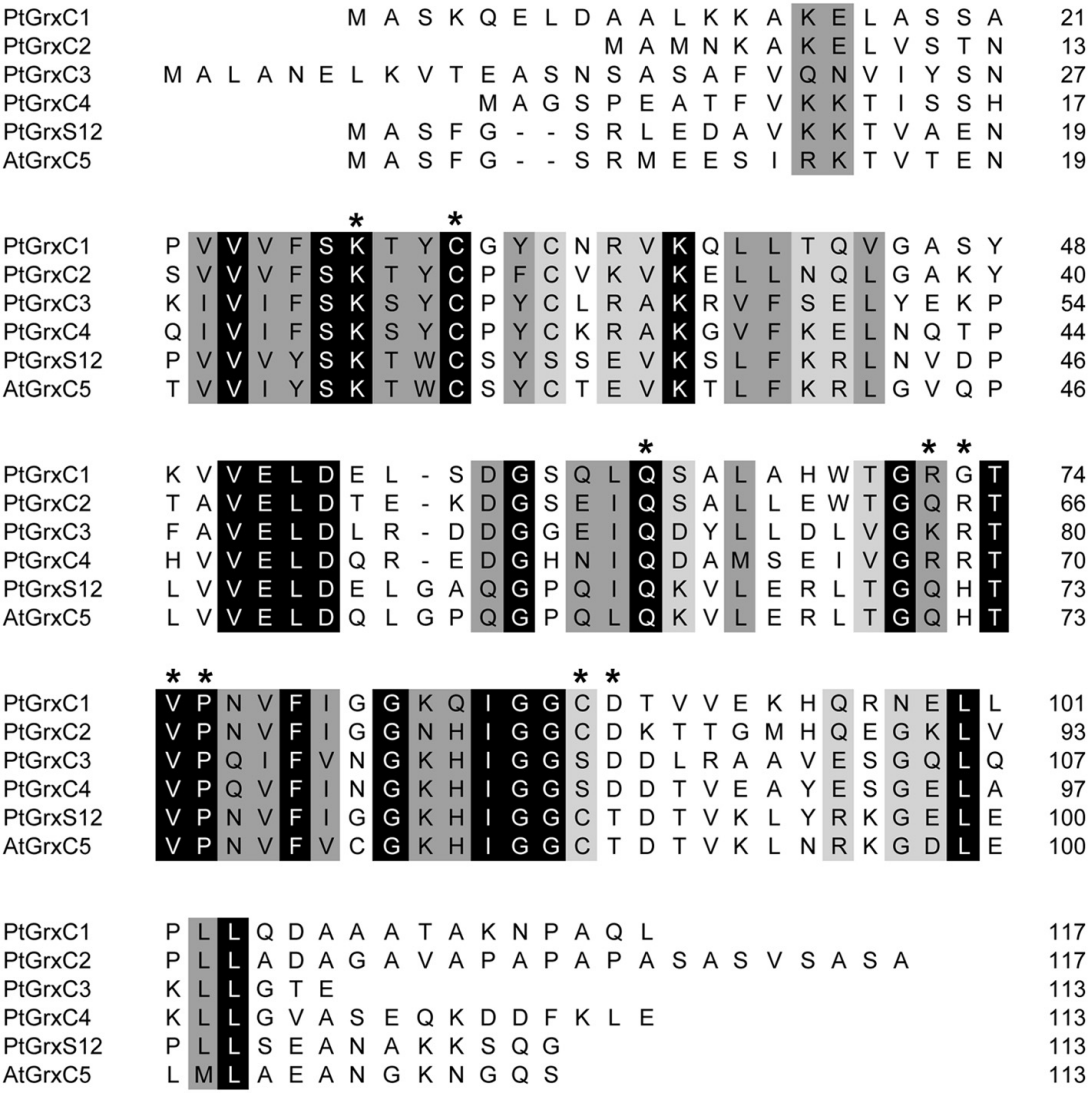
**Amino acid alignment of class I glutaredoxins**. The alignment was done using ClustalW from sequences of the recombinant proteins that are deprived of putative targeting sequences. The strictly conserved amino acids are depicted in white on black. Other conservative amino acid changes are indicated in black on gray. Residues involved in GSH binding based on the 3D structures of poplar GrxC4 and GrxS12 and Arabidospis GrxC5 are marked by an asterisk.

An amino acid sequence alignment of the predicted mature forms of all poplar class I Grxs reveals several conserved residues, particularly those around the active sites and those involved in glutathione binding (Figure 1). However, GrxC1 and GrxC2 present CGYC and CPFC active site motifs, respectively, different from the CPYC active site found in GrxC3 and GrxC4. Interestingly, contrary to GrxC3 and GrxC4, GrxC1 and GrxC2 isoforms possess an additional cysteine (Cys_*C*_) located in their C-terminal part in a IGGCD motif, as in human Grx1, *E. coli* Grx3 and the two other class I Grxs found in plants, GrxC5 and GrxS12 (Figure 1). With the aim of comparing the redox and kinetic properties of all dithiol Grxs (GrxC5 is not found in poplar) and understanding why these duplicated genes have been retained during evolution, we have performed a thorough biochemical analysis of the corresponding recombinant proteins.

The catalytic properties of each isoform were first determined by measuring their reductase activity using the classical HED and DHA assays, which measure the reduction of glutathionylated-β-mercaptoethanol or of dehydroascorbate, respectively. All proteins display a better catalytic efficiency (*k*_*cat*_/*K*_*m*_) in the HED test with a difference of two orders of magnitude (Table 1). In both assays, the kinetic parameters determined for each protein follow the same tendency. GrxC3 and C4 have quite comparable turnover numbers, with *k*_*cat*_ values about 10 fold higher than those of GrxC1 and C2. In terms of catalytic efficiencies, the dichotomy between GrxC1/C2 and GrxC3/C4 is clear but it is often below this factor of 10 because GrxC3 and C4 have higher apparent *K*_*m*_ values for both substrates. Overall, GrxC1 always exhibited the lowest catalytic efficiency and GrxC4 the highest. Concerning the apparent affinity for GSH, although there are differences in the *K*_*m*_ values for GSH between the four isoforms, ranging from ~2.0 mM for GrxC4 to ~3.9 mM for GrxC2, the GrxC1/C2 and GrxC3/C4 dichotomy is not visible using this parameter.

**Table 1:** **Kinetic parameters of poplar dithiol class I glutaredoxins**.

		**PtGrxC1**	**PtGrxC2**	**PtGrxC3**	**PtGrxC4**	**PtGrxS12^a^**	**AtGrxC5^a^**
β-ME-SG	*K*_*m*_ (mM)	0.10 ± 0.01	0.06 ± 0.01	0.50 ± 0.06	0.32 ± 0.04	0.31 ± 0.04	0.20 ± 0.02
	*k*_*cat*_(s^−1^)	7.9 ± 0.2	11.7 ± 0.1	77.0 ± 4.4	90.3 ± 4.9	23.1 ± 0.9	1.21 ± 0.03
	*k*_*cat*_/*K*_*m*_(x 10^5^ M^−1^.s^−1^)	0.79	1.95	1.54	2.82	0.745	0.06
DHA	*K*_*m*_ (mM)	0.55 ± 0.06	0.37 ± 0.03	1.14 ± 0.13	0.60 ± 0.09	0.38 ± 0.19	0.21 ± 0.03
	*k*_*cat*_(s^−1^)	0.6 ± 0.1	1.3 ± 0.1	6.5 ± 0.4	8.2 ± 0.6	1.7 ± 0.2	0.23 ± 0.01
	*k*_*cat*_/*K*_*m*_(x 10^3^ M^−1^.s^−1^)	1.09	3.51	5.70	13.67	4.6	1.1
GSH	*K*_*m*_ (mM)	2.76 ± 0.26	3.87 ± 0.53	3.60 ± 0.42	1.96 ± 0.28	4.0 ± 0.5	nd
	*k*_*cat*_(s^−1^)	13.8 ± 0.6	30.1 ± 2.3	131.3 ± 8.3	102.9 ± 6.5	92.8 ± 5.3	nd
	*k*_*cat*_/*K*_*m*_(x 10^3^ M^−1^.s^−1^)	5.00	7.78	36.47	52.50	23.20	nd

The apparent K_m_ values for β-ME-SG (glutathionylated β-mercaptoethanol) were determined using a HED concentration range of 0.1–1 mM in presence of 2 mM GSH and 100 nM enzyme for GrxC1, 50 nM for GrxC2, 10 nM for GrxC3 and GrxC4. The apparent K_m_ values for DHA were determined using a DHA concentration range of 0.1–1 mM in presence of 2 mM GSH and 2 μM enzyme for GrxC1, 1 μM for GrxC2, 200 nM for GrxC3, and 150 nM for GrxC4. The apparent K_m_ values for GSH were determined using a GSH concentration range of 0.25–5 mM in the presence of 0.7 mM HED and 100 nM enzyme for GrxC1, 50 nM for GrxC2, 10 nM for GrxC3 and GrxC4. The k_cat_ and K_m_ values were calculated by non-linear regression using the Michaelis–Menten equation. Values are the means ± SD of three replicates.

a Data extracted from the following references Couturier et al. (2009b, 2011), Zaffagnini et al. (2012a). nd, not determined.

**Figure 2:**
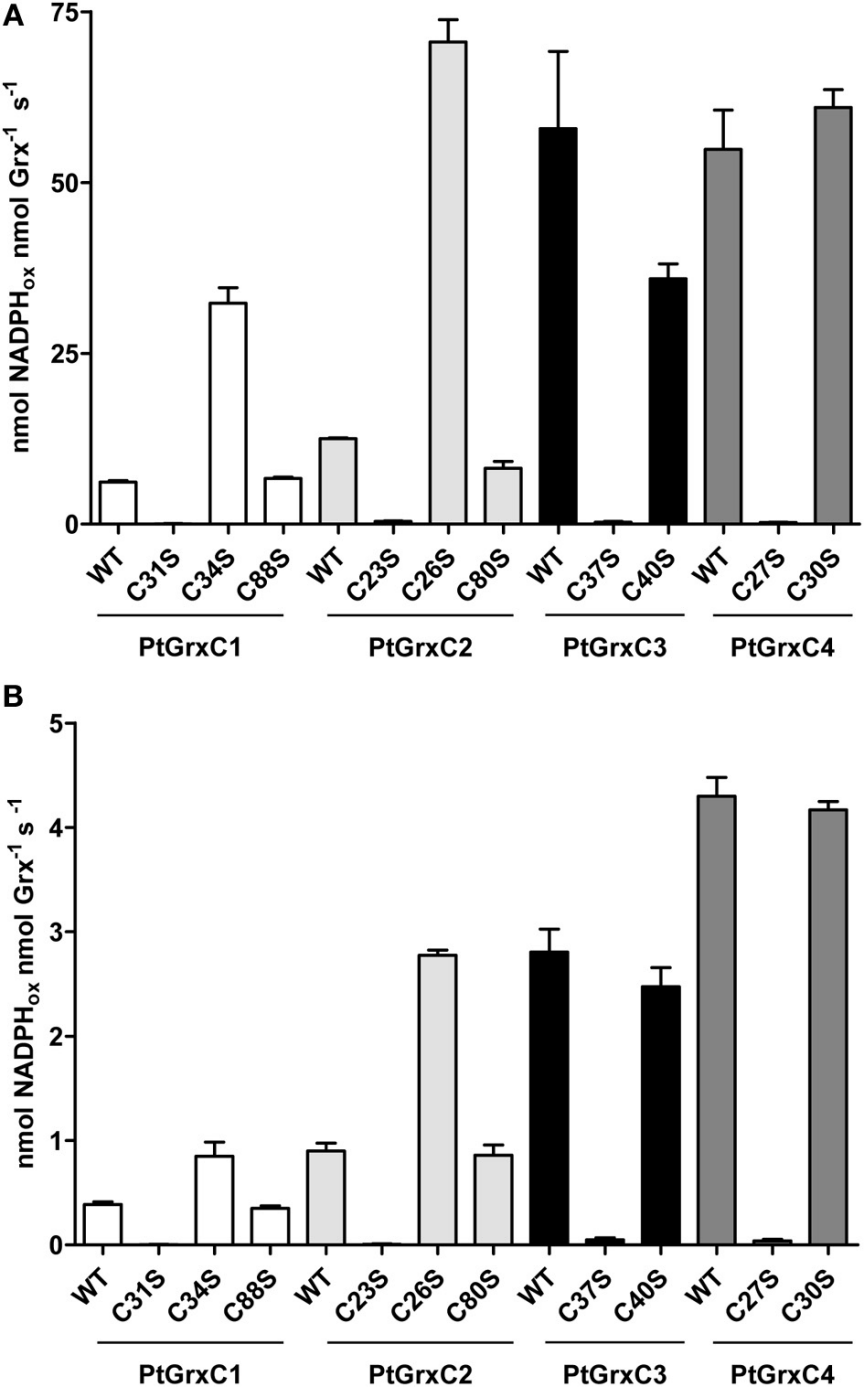
**Reductase activity of GrxC1, C2, C3, and C4 and of cysteine-to-serine variants. (A)** HED reduction was measured in the presence of 2 mM GSH and 0.7 mM HED using either 100 nM GrxC1, 20 nM for GrxC1 C34S, 50 nM GrxC2, 10 nM for GrxC2 C26S, 10 nM GrxC3, or 10 nM GrxC4. **(B)** DHA reductase activity was measured in the presence of 2 mM GSH and 1 mM DHA using either 2 μM GrxC1, 1 μM for GrxC1 C34S, 1 μM GrxC2, 250 nM GrxC2 C26S, 200 nM GrxC3, or 150 nM GrxC4. The data are expressed as nmol NADPH oxidized per nmol enzymes per s and represent means ± SD of three separate experiments.

Then, the involvement for the catalytic mechanism of the different cysteine residues present in each isoform was investigated by comparing the activity of WT proteins to their corresponding cysteinic mutants using similar assays at fixed substrate concentrations (Figure 2). As expected, all monocysteinic mutants for the catalytic cysteine Cys_A_ (GrxC1 C31S, GrxC2 C23S, GrxC3 C37S, and GrxC4 C27S) were inactive, confirming that it is indispensable for the reductase activity. On the contrary, mutating Cys_*C*_, in positions 88 and 80 in GrxC1 and GrxC2, respectively, did not affect catalytic activity. Finally, the mutation of Cys_B_ has a different impact depending on the isoforms. The protein variants for this cysteine in GrxC1 and C2 (GrxC1 C34S and GrxC2 C26S) were 5 fold more efficient and 2–3 fold more efficient than the corresponding WT proteins in the HED and DHA assays, respectively. On the other hand, the mutation of this cysteine in GrxC3 and GrxC4 has almost no impact, since, only a slight decrease was observed in the HED assay for the GrxC3 C40S variant.

### Oxidative modifications of cysteine residues

Several observations prompted us to analyze the sensitivity of all dithiol class I Grxs to oxidizing treatments and the resulting changes in their redox state. First, the mutation of Cys_B_ increased the catalytic efficiency of GrxC1 and C2 but not of GrxC3 and C4 suggesting possible difference in the reaction mechanisms. Second, although Cys_*C*_ does not influence their catalytic activity, the migration of the purified proteins in non-reducing SDS PAGE gels indicated the formation of covalent linkages for GrxC1 and C2 that are not visible for GrxC3 and C4 (data not shown).

**Figure 3:**
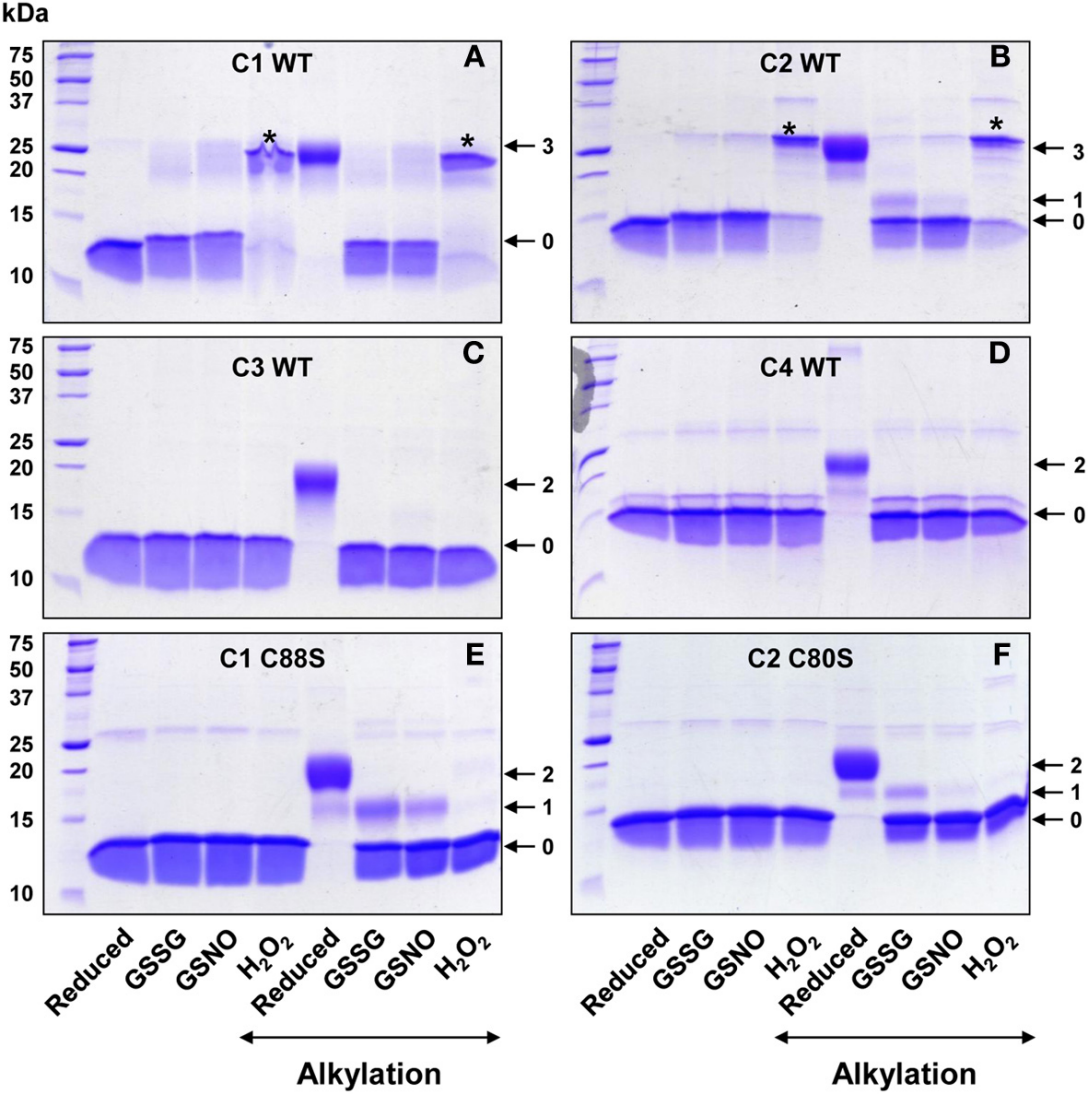
**Sensitivity of poplar Grxs to oxidative treatments**. Pre-reduced Grxs were subjected to 2 h oxidative treatments with H_2_O_2_, GSSG, and GSNO as described in the “materials and methods” section. Prior to separation on SDS-PAGE gels in non-reducing conditions, GrxC1 WT **(A)**, GrxC2 WT **(B)**, GrxC3 WT **(C)**, GrxC4 WT **(D)**, GrxC1 C88S **(E)**, and GrxC2 C80S **(F)** were alkylated or not with 2 kDa mPEG maleimide. The shift observed following the alkylation of the free thiol groups is larger than expected (ca. 4 kDa instead of 2 kDa), but this has been already observed previously (Chibani et al., 2011). The numbers on the right correspond to the number of alkylated thiols that remained free after reducing or oxidizing treatment of Grxs. The stars in panels **(A)** and **(B)** indicate the position of covalent dimer.

For this purpose, reduced proteins have been treated for 2 h with a 10 fold excess of GSSG or H_2_O_2_, or a 5 fold excess of GSNO. The resulting redox state was analyzed by mass spectrometry and by protein migration on non-reducing SDS PAGE with or without thiol alkylation by 2 kDa mPEG maleimide, which allows distinguishing reduced and oxidized forms of Grxs. Since reduced mPEG-alkylated proteins and covalent dimers of GrxC1 and GrxC2 migrate roughly at the same position, controls without alkylation have been included (Figure 3). With non-alkylated proteins, we confirmed that an H_2_O_2_ treatment, but not the GSSG and GSNO treatments, promoted the formation of covalent dimers of GrxC1 and GrxC2 (Figures 3A,B) but not of GrxC3 and GrxC4 (Figures 3C,D). Only a small part of GrxC2 dimer is visible after these 2 h GSSG and GSNO treatments. From the procedure that included an alkylation step, we can further conclude that all treatments led essentially to a complete oxidation of all thiol groups present on all Grxs since almost no shift of migration was observed. A minor form with one free thiol group is visible for GrxC2 after a GSSG treatment. It is even more discrete upon GSNO treatment. For GrxC3 and C4, these oxidized monomeric forms are interpreted as containing intramolecular disulfides between active site cysteines (Cys_A_-Cys_B_) (Figures 3C,D). For GrxC1 and C2, these two cysteines should also form this intramolecular disulfide, and Cys_*C*_ is either modified by a glutathione or a nitroso (in the case of GSNO) adduct as confirmed by mass spectrometry analyses (see below) or it is involved in an intermolecular disulfide upon H_2_O_2_ treatment. From similar experiments performed with the GrxC1 C88S and GrxC2 C80S mutants (Figures 3E,F), the absence of covalent dimers indicated that Cys_*C*_ is indeed involved in dimerization. Moreover, in the context of these variants, after GSSG and GSNO treatments, the proteins mainly contain an active site disulfide bond whereas a small part of the proteins has one remaining free thiol, likely corresponding to glutathionylated intermediates.

In order to identify the type of protein modification formed by these oxidative treatments, all proteins have been analyzed by mass spectrometry (Table 2 and Figures S1–S6). First, all reduced proteins gave a single species with a mass decrease of *ca* 131 Da compared to the calculated theoretical molecular masses. It does undoubtedly correspond to the cleavage of the first methionine as expected from the presence of an alanine in second position. The results obtained with GrxC3 and GrxC4 are more easily interpreted since these proteins contain only two cysteines. In the presence of H_2_O_2_, and in accordance with the absence of detected free thiols in the previous experiments (Figures 3C,D), GrxC3 and GrxC4 are exclusively detected as a monomeric oxidized form with a decrease of ~2 Da confirming the formation of a Cys_A_-Cys_B_ intramolecular disulfide bond. The same forms were primarily obtained upon GSSG and GSNO treatments but a minor monomeric form with a mass increment of 305 Da corresponding to one glutathione adduct was also detected (Table 2). It was not detected on gels presumably because of the presence of minute amounts. Nevertheless, this suggests that the glutathionylated form represents an intermediate for the formation of the intramolecular disulfide.

**Table 2:** **Electrospray ionization mass spectrometry analysis of the redox states of poplar class I Grxs**.

**Protein**	**Theoretical size (Da)**	**Theoretical size without Met (Da)**	**DTT_red_ treatment**	**GSSG treatment**	**Relative percentage values (%)**	**GSNO treatment**	**Relative percentage values (%)**	**H_2_O_2_ treatment**	**Relative percentage values (%)**	**Deduced redox state**		
										**Cys_A_**	**Cys_B_**	**Cys_C_**
PtGrxC1 WT	12,514.3	12,383.1	12,382.7	**12,686.3 (+303.6)**	**76.4**	12,380.0 (−2.7)	5.7	**12,412.2 (+29.5)**	**72.1**	SH	SH	SH
				12,993.8 (+611.1)	23.6	**12,686.3 (+303.6)**	**81.8**	24,756.5 (×2)	27.9			
						12,993.4 (+610.7)	12.5					
										S-S		SH
										S-S		SO_2_H
										S-S		SSG
										SSG	SH	SSG
										S-S		S-S (interchain)
PtGrxC2 WT	12,178.9	12,047.7	12,047.3	12,045.0 (−2.3)	11.1	12,044.9 (−2.4)	23.7	12,077.2 (+32.2)	50	SH	SH	SH
				**12,351.5 (+304.2)**	**68.4**	**12,350.7 (+303.4)**	**76.3**	24,088.9 (×2)	50			
				12,657.9 (+610.6)	20.5							
										S-S		SH
										S-S		SO_2_H
										S-S		SSG
										SSG	SH	SSG
										S-S		S-S (interchain)
PtGrxC3 WT	12,516.2	12,385.0	12,384.7	**12,382.7 (−2.0)**	**66.3**	**12,382.9 (−1.8)**	**75.9**	12,382.7 (−2.0)	100	SH	SH	
				12,690.0 (+305.3)	33.7	12,690.0 (+305.3)	24.1					
										S-S		
										SSG	SH	
PtGrxC4 WT	12,526.1	12,394.9	12,394.4	**12,392.0 (−2.4)**	**62.5**	**12,392.1 (−2.5)**	**90.8**	12,392.3 (−2.7)	100	SH	SH	
				12,699.6 (+305.2)	37.5	12,699.2 (+304.8)	9.2					
										S-S		
										SSG	SH	
PtGrxC1 C88S	12,498.2	12,367.0	12,366.6	**12,364.7 (−1.9)**	**64.0**	**12,364.5 (−2.1)**	**79.6**	12,364.6 (−2.0)	100	SH	SH	
				12,671.8 (+305.2)	36.0	12,672.0 (+305.4)	20.4					
										S-S		
										SSG	SH	
PtGrxC2 C80S	12,162.8	12,031.7	12,030.6	**12,029.3 (−1.3)**	**81.5**	12,028.8 (−1.8)	100	12,028.7 (−1.9)	100	SH	SH	
				12,336.5 (+305.9)	18.5							
										S-S		
										SSG	SH	

Reduced proteins and proteins treated with GSNO, GSSG, or H_2_O_2_ were analyzed by mass spectrometry. The mass differences are indicated between parentheses. The mass accuracy is generally ±0.5 Da. Comparing mass differences and migration profiles of corresponding alkylated proteins under non-reducing conditions enabled to deduce the redox state of each cysteine residue. When several oxidized forms are detected and with clear different abundance, the major form is indicated in bold. The relative percentage of each form has been deduced from the spectra assuming equal ionization of all GRX forms.

The results obtained for GrxC1 and GrxC2 are slightly more complex. In response to an H_2_O_2_ treatment, two different species are formed for both proteins. In accordance with the results from alkylation experiments, a major form corresponds to a disulfide-bridged dimer having also an intramolecular Cys_A_-Cys_B_ disulfide bond. The detected monomeric forms, which have no free thiols or a very small fraction as observed for GrxC2, exhibited a mass increment of 29.5 Da for GrxC1 and 32.2 Da for GrxC2, suggesting the presence of two oxygen atoms. Considering that this overoxidation is not observed for GrxC1 C88S and GrxC2 C80S variants which only form monomeric proteins with an intramolecular disulfide (as indicated by the mass decrease of 2 Da), the results obtained for the WT proteins are best interpreted as the formation of an intramolecular disulfide between the active site cysteines (Cys_A_-Cys_B_) and of a sulfinic acid on Cys_*C*_. In the presence of GSSG or GSNO, GrxC1 and GrxC2 exist only under monomeric forms but with variable oxidation states. Except for the GSSG-treated GrxC1, there is a minor form with a mass decrease of about 2 Da consistent with the formation of an intramolecular disulfide. Another minor form, found in all samples except the GSNO-treated GrxC2, exhibited a mass increment of 610.5–611 Da, which corresponded to the presence of two glutathione adducts. However, the major species formed by these two treatments is a monomer with a mass increment of about 303.5 Da which is consistent with the presence of one glutathione adduct on Cys_*C*_ in addition to the Cys_A_-Cys_B_ disulfide bond. Accordingly, the major species detected in protein variants mutated for Cys_*C*_ (Grx C1 C88S and GrxC2 C80S) is the one with an intramolecular disulfide (decrease of 2 Da upon GSSG or GSNO treatments). The glutathionylated forms detected by mass spectrometry were visible on gels (see Figures 3E,F).

**Figure 4:**
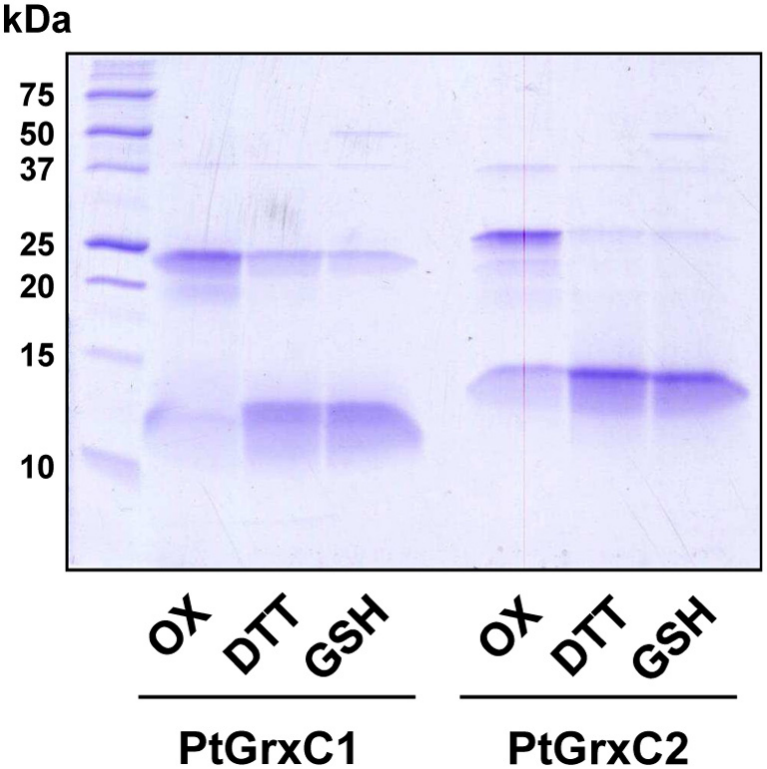
**GSH-dependent reduction of the intermolecular disulfide in poplar GrxC1 and GrxC2**. The ability of GSH to reduce poplar disulfide-bridged dimer GrxC1 and disulfide-bridged dimer GrxC2 generated by a 2 h H_2_O_2_ treatment was evaluated in the presence of 1 mM DTT and 1 mM GSH coupled to NADPH and GR. The lanes Ox represent the initial oxidized proteins. Proteins were separated on SDS-PAGE gels under non-reducing conditions.

The *in vitro* observation of intermolecular disulfide bonds in GrxC1 and C2, involving Cys_*C*_, raised the question of their possible reduction. Thus, we tested the ability of DTT but also of a physiological GSH reducing system composed of NADPH, GR, and GSH to reduce this intermolecular disulfide, the formation of which was initiated by a 2h H_2_O_2_ treatment. As shown in Figure 4, both DTT and GSH are able to reduce GrxC1 and GrxC2 dimers to a similar extent. Whether minute amounts of reduced monomeric Grx initially present or formed can catalytically increase the reduction of dimeric Grxs in the presence of GSH cannot be excluded. Overall, it indicates that the formation of these disulfide-bridged forms of GrxC1 and GrxC2 isoforms is reversible, notably by GSH, a relevant cellular physiological reductant.

### Redox properties of class I glutaredoxins

**Figure 5:**
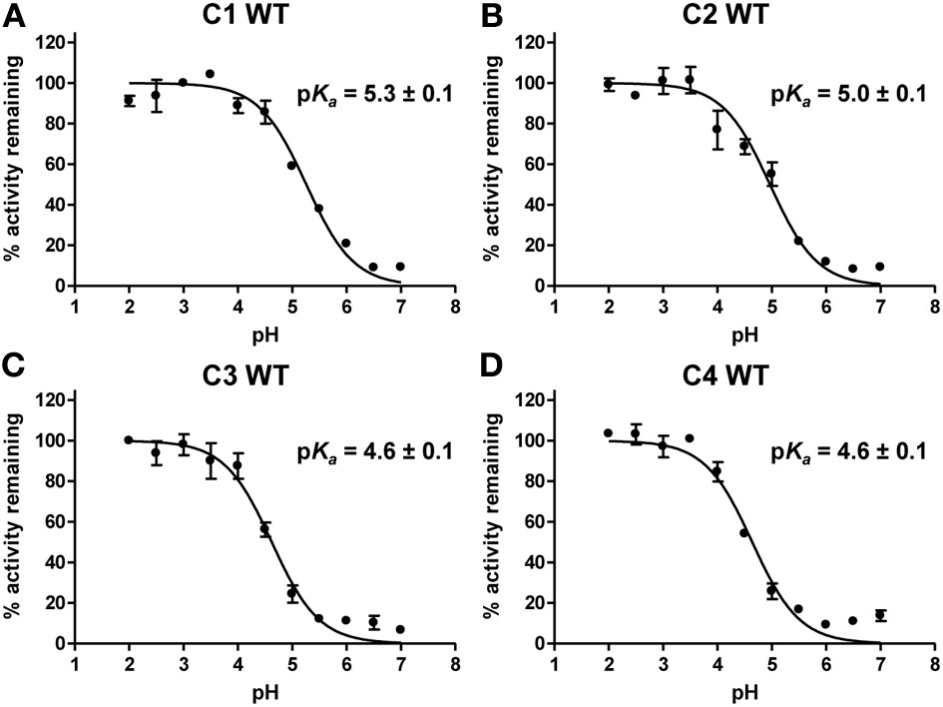
**p*K*_*a*_ determination of the catalytic cysteine of poplar GrxC1, C2, C3, and C4**. Reduced GrxC1 **(A)**, GrxC2 **(B)**, GrxC3 **(C)**, and GrxC4 **(D)** WT proteins were incubated in different buffers ranging from pH 2.0 to 7.0 in presence or not of iodoacetamide (IAM) prior to measurement of their activity by using the HED assay. The percentages of remaining activity at each pH were determined by comparing the activity of the enzyme incubated with and without IAM and an adaptation of the Henderson–Hasselbach equation was used for p*K*_*a*_ value determination. The data are represented as mean ± SD of three separate experiments.

As in other thiol-dependent oxidoreductases, the catalytic efficiency of class I Grxs should be at least partially governed by thermodynamic parameters as the redox midpoint potentials of dithiol-disulfide couples and by the reactivity of the catalytic cysteine which is dependent on its p*K*_*a*_. Hence, we determined the Cys_A_ p*K*_*a*_ of the four Grx isoforms using a method relying on iodoacetamide which is an alkylating reagent reacting with thiolates but not thiols. The pH-dependent inactivation of WT Grx by iodoacetamide was followed by comparing the activity of WT proteins in the HED assay, after pre-incubation of the protein in different buffers ranging from pH 2.0 to 7.0 in the presence or not of iodoacetamide. From these titration curves, we obtained p*K*_*a*_ values of 5.3 ± 0.1 for GrxC1, 5.0 ± 0.1 for GrxC2, 4.6 ± 0.1 for GrxC3, and 4.6 ± 0.1 for GrxC4 (Figure 5). These results indicate first that at physiological pH, the thiolate ion will be the dominant species for all Grxs. Although the differences are not huge, it also confirmed the existence of two subgroups, GrxC1 and C2 having higher p*K*_*a*_compared to GrxC3 and C4.

**Figure 6:**
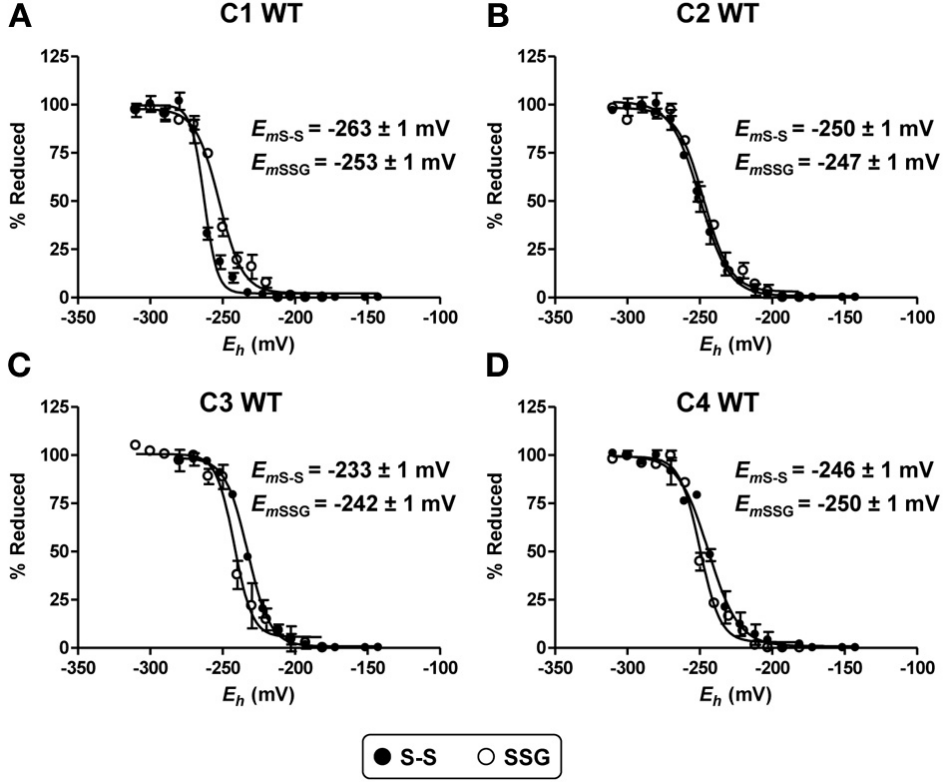
**Determination of redox potential of oxidized poplar GrxC1, C2, C3, and C4**. Representative curve of oxidation-reduction titration of Cys_A_-Cys_B_ intramolecular disulfide forms (black circles) and glutathionylated Cys_A_ (white circles) forms of poplar GrxC1 **(A)**, GrxC2 **(B)**, GrxC3 **(C)**, and GrxC4 **(D)** at pH 7.0. The titration was carried out using a total DTT or GSH concentration of 2 mM for 2 h and resulting free thiol groups were labeled by mBBr. Values in the text are the means ± SD of three replicates.

Two disulfide bond forms may be catalytically relevant for all these Grxs, the one with a glutathionylated Cys_A_ and the one with a Cys_A_-Cys_B_ intramolecular disulfide bond. For measuring the midpoint redox potentials (*E*_*m*_) of each of these disulfide/dithiol couples, we used different protein variants and two distinct oxidative treatments in order to discriminate between these two oxidized forms. To obtain proteins containing an intramolecular disulfide bond, reduced Grxs possessing only the two active site cysteines (GrxC1 C88S, GrxC2 C80S, GrxC3 WT, and GrxC4 WT) have been treated with H_2_O_2_ before desalting on G25 columns. On the other hand, glutathionylated proteins have been obtained by treating reduced proteins in which only Cys_A_ was remaining (GrxC1 C34/88S, GrxC2 C26/80S, GrxC3 C40S, and GrxC4 C30S) with GSNO before desalting. The *E*_*m*_ values determined at pH 7.0 for the intramolecular disulfide range from −233 mV for GrxC3 to −263 mV to GrxC1 (Figure 6). On the other hand, only little variations were visible when measuring *E*_*m*_ values for the glutathione adduct formed on the catalytic cysteine. They varied from −242 mV for GrxC3 to −254 mV for GrxC1. Overall, the intramolecular active site disulfide bond and glutathionylated forms of GrxC2 and GrxC4 present similar *E*_*m*_ values. A 10 mV difference was measured between both forms for GrxC1 and C3, the more electronegative redox potential being the active site disulfide in GrxC1 and the glutathione adduct in GrxC3. However, the differences are small and should not explain the observed differences in catalytic efficiencies. In reducing cellular environments, all these oxidation forms should be compatible with glutathione reduction. Considering their possible targeting to secretory pathways, this may not be the case for Grx3 and C4 if they are in the endoplasmic reticulum or in the apoplasm.

## Discussion

### Duplication and neo-functionalization into class I glutaredoxins

Among all living organisms, higher plants have the highest number of genes coding for Grxs. In addition to specific isoforms such as those belonging to the class III, land plants also possess an expanded class I, usually with 5–6 isoforms (Couturier et al., 2009a). From previous phylogenetic analyses, it was quite clear that, there are three Grx couples, GrxC1/C2, GrxC3/C4, and GrxC5/S12 that probably evolved by more or less recent duplication (Couturier et al., 2009a). Interestingly, GrxC1 and C2 proteins are cytoplasmic proteins (Riondet et al., 2012), GrxC3 and C4 have N-terminal extensions predicted to target the proteins to secretory pathways but the final destination is not known, and GrxC5 and S12 are chloroplastic (Couturier et al., 2009b, 2011). The case of GrxC1 and C2 is interesting. Whereas GrxC2 prototypes are found in all land plants (mosses, lycophytes, monocots, dicots), GrxC1 is specifically found in dicots. A redundancy between both isoforms is suggested in *A. thaliana* by the observation that single knock-out mutants in *grxc1* or *grxc2* are aphenotypic, whereas a double mutant is lethal at an early stage after pollinization (Riondet et al., 2012). Considering that GrxC1, but not GrxC2, is able to bind an Fe-S cluster (at least *in vitro*), this would rather suggest that the essential nature of these two paralogs is linked to their reductase activity. These results point to the need of investigating at the genetic level a possible redundancy among the GrxC3/C4 and GrxC5/12 couples. This study primarily focused on the biochemical properties of dithiol class I Grxs since a detailed structure-function analysis was previously achieved on the two other prototypes of class I Grxs, the poplar monothiol GrxS12 and the Arabidopsis dithiol GrxC5 (Couturier et al., 2009b, 2011). GrxC5 isoforms are only found in species of the Brassicaceae family. We have demonstrated in particular that, although being very similar in sequence due to their recent duplication, a single substitution in the active site sequence (from WCSYS in GrxS12 to WCSYC in GrxC5) explains the capacity of GrxC5, but not GrxS12, to bind an [Fe_2_-S_2_] cluster into a dimer. Among other class I Grxs, only GrxC1 can also exist under a holoform binding an Fe-S cluster (Rouhier et al., 2007). In this case, the mutational analysis showed that the presence of the glycine in the CGYC active site instead of a proline in other Grxs (CPFC in GrxC2 or CPYC in GrxC3 and C4) was sufficient to explain this property. Overall, this indicates that subtle sequence differences affecting key residues may provide different properties to otherwise quite close proteins. This prompted us to further analyze the various glutaredoxins of this class as GrxC2 and GrxC3 have been only poorly studied so far.

### Two subgroups in dithiol Grxs exhibit distinct kinetic properties

Considering the divergence observed in the amino acid sequences, the first aim of this work was to perform a thorough comparative analysis of the biochemical properties of GrxC1, GrxC2, GrxC3, and GrxC4 from poplar. All these proteins exhibit deglutathionylation or reductase activities using classical HED and DHA assays with catalytic efficiencies (10^5^ M^−1^ s^−1^ for HED and 10^3^–10^4^ M^−1^ s^−1^ for DHA) in the same range as those previously reported for plant and non-plant class I Grxs (Table 3). Two subgroups can however be distinguished, GrxC1 and GrxC2 appeared to be less efficient catalysts than GrxC3 and GrxC4. The difference is particularly clear when considering the turnover numbers (k_cat_) and the k_cat_/*K*_DHA_ and k_cat_/*K*_GSH_. This is not visible with k_cat_/*K*_β −*ME*−*SG*_ because the differences in the turnover number are compensated in this case by differences in apparent affinity in favor of GrxC1 and C2. An observation that may be interesting to understand these differences is that GrxC1/C2 and GrxC3/C4 should have quite different charge distribution at the protein surface that could account for substrate recognition and thus for the observed differences in catalytic efficiency. Indeed, their behavior on ion exchange chromatography indicates that GrxC1 and GrxC2 should be more basic having a pI close or higher than 8, whereas GrxC3 and GrxC4 should be more acidic having a pI value below 8 (theoretical pI estimations are indeed comprised between 5 and 6).

**Table 3:** **Kinetic and biochemical parameters of selected plant and non-plant class I Grxs**.

**Name**	**Active site sequence**	**HED**			**DHA**			**GSH**			**p*K*_*a*_**	***E*_*m*_ (pH7.0) (mV)**	**References**
		***K*_*m*_ (mM)**	**k_*cat*_ (s^−1^)**	***k_cat_/K_m_* (M^−1^ s^−1^)**	***K*_*m*_**	***k*_*cat*_**	***k*_*cat*_/*K*_*m*_**	***K*_*m*_**	***k*_*cat*_**	***k_cat_/K_m_***			
AtGrxC1	YCGYC	0.74 ± 0.01	38.91 ± 0.43	5.3 × 10^4^	0.08 ± 0.01	3.03 ± 0.82	3.8 × 10^4^	nd	nd	nd	nd	nd	Riondet et al., 2012
AtGrxC2	YCPYC	0.34 ± 0.02	18.44 ± 0.57	5.4 × 10^4^	0.08 ± 0.01	3.26 ± 0.03	4.1 × 10^4^	nd	nd	nd	nd	nd	Riondet et al., 2012
ScGrx1	YCPYC	0.14	3.5	2.5 × 10^4^	nd	Nd	nd	6.2	17.1 (HED)	2.75 × 10^3^	3.2 to 4.0 ± 0.2	nd	Discola et al., 2009
ScGrx2	YCPYC	0.6	85	1.4 × 10^5^	nd	nd	nd	0.9	129 (HED)	1.43 × 10^5^	3.1 to 3.5 ± 0.2	nd	Discola et al., 2009
CrGrx1	HCPYC	0.34 ± 0.04	30.40 ± 4.31	8.9 × 10^4^	0.39 ± 0.03	1.67 ± 0.09	4.3 × 10^3^	2.6 ± 0.5	161.6 ± 15.0 (HED)	6.1 × 10^4^	3.9 ± 0.1	nd	Zaffagnini et al., 2008
CrGrx2	YCPYC	0.20 ± 0.04	7.10 ± 0.27	3.5 × 10^4^	0.17 ± 0.05	0.84 ± 0.06	4.9 × 10^3^	3.7 ± 0.7	26.5 ± 4.1 (HED)	7.1 × 10^3^	4.8 ± 0.1	nd	Gao et al., 2010
HsGrx1	TCPYC	1.07	8.16	7.6 × 10^3^	nd	Nd	nd	2.2	293 (HED)	1.4 × 10^5^	3.6	−232	Johansson et al., 2004; Jao et al., 2006; Sagemark et al., 2007
HsGrx2	SCSYC	0.11	1.3	1.2 × 10^4^	nd	Nd	nd	5.9	71.3 (HED)	1.2 × 10^4^	4.6	−221	Jao et al., 2006; Sagemark et al., 2007; Gallogly et al., 2008

nd, not determined.

In order to further explain the differences between poplar class I Grxs, we determined the p*K*_*a*_ value of their catalytic cysteine, as it was proposed that reactivity of human and Chlamydomonas class I Grxs is partially related to the p*K*_*a*_ of N-terminal active site cysteine (Gallogly et al., 2008; Gao et al., 2010). The values obtained for poplar Grxs are globally close to those reported for other plant and non-plant class I Grxs (Table 3), including the value of 3.9 determined for poplar GrxS12 using the same procedure (Zaffagnini et al., 2012a; Roos et al., 2013). Interestingly, a difference of ca 0.5 unit is visible between GrxC1/C2 (p*K*_*a*_ around 5) and GrxC3/C4 (p*K*_*a*_ around 4.6). Such differences between paralogs have been described already since *C. reinhardtii* Grx1, *S. cerevisiae* Grx2, and human Grx1 exhibit higher turnover numbers often translated into higher catalytic efficiencies compared to *C. reinhardtii* Grx2, *S. cerevisiae* Grx1, and human Grx2, respectively (Table 3). For the human and *C. reinhardtii* enzymes, it is also associated to a difference in p*K*_*a*_ values of *ca* 1 unit, the enzymes with the lowest p*K*_*a*_ being the most efficient. According to the Brønsted theory, in a thiol-disulfide exchange reaction, each unit decrease in the p*K*_*a*_ value of the leaving group is responsible for a 4 fold increase in the second-order rate constant (Szajewski and Whitesides, 1980). This could fit with the conclusion that differences in p*K*_*a*_ values between class I poplar Grx isoforms are at least partially responsible for the differences in catalytic efficiencies observed. On the other hand, only small differences were observed for the oxidation-reduction midpoint potentials of both the glutathione adduct or the intramolecular disulfide and they are not discriminating GrxC1/C2 vs. GrxC3/C4. The measured values (from −230 to −260 mV) are in the same range than those reported for *E. coli* Grx1 (−233 mV) and human Grx1 and 2 (−232 and −221 mV) (Table 3) (Aslund et al., 1997; Sagemark et al., 2007).

### The role of the recycling Cys_B_: monothiol or dithiol reaction mechanism?

Interestingly, the differences in protein activities between GrxC1 or C2 and GrxC3 or C4 are attenuated when Cys_B_ of GrxC1 or C2 is removed (see Figure 2). This has been reported also for Arabidopsis GrxC5, yeast Grx1, human Grx1 and Grx2, and pig Grx (Yang and Wells, 1991; Yang et al., 1998; Johansson et al., 2004; Gallogly et al., 2008; Discola et al., 2009; Couturier et al., 2011). In clear contrast, mutating this cysteine in yeast Grx2, *E. coli* Grx1 and Grx3 led to a decrease of Grx activity (Bushweller et al., 1992; Nordstrand et al., 1999; Peltoniemi et al., 2006; Discola et al., 2009; Saaranen et al., 2009). For *E. coli* Grx1 and by extension for Grxs for which mutation of this cysteine decreased their activity, this decrease was attributed to a change in glutathione recognition, binding, or affinity, because Cys_B_ would determine the glutathione specificity of the glutathionylated Grx reduction step (Saaranen et al., 2009). For GrxC1 and GrxC2 and other eukaryote Grxs mentioned above in which the mutation of this cysteine increased their activity, the data indicates that the presence of Cys_B_ slows down the reaction for some reason. It may eventually modify the p*K*_*a*_ of Cys_A_. This has been experimentally confirmed for a pig Grx, for which the p*K*_*a*_ of the catalytic Cys22 is 3.8 in a WT protein but 4.9 and 5.9 in C25S and C25A variants (Yang and Wells, 1991). Another possibility is that a Cys_A_-Cys_B_ intramolecular disulfide is formed during activity measurements either because it constitutes a regular step of the reaction cycle in some situations, especially for dithiol substrates or because it is a side reaction reflecting the competition between Cys_B_ and GSH for the reduction of the glutathionylated Cys_A_ (Gallogly et al., 2009). Then, the reduction of this Cys_A_-Cys_B_ intramolecular disulfide intermediate would require two molecules of GSH vs. one molecule of GSH for the glutathionylated form and add two steps in the reaction mechanism. Thus, it seems unfavorable in terms of efficiency and energetic cost.

We have previously analyzed possible variations in the redox state of the dithiol AtGrxC5 (WCSYC active site) in response to 15 min treatments with GSSG or oxidized DTT by measuring the tryptophan intrinsic fluorescence of the protein, since the oxidation of the catalytic cysteine quenched the fluorescence of the adjacent tryptophan (Couturier et al., 2011). It was concluded that the dithiol AtGrxC5 cannot form a Cys_A_-Cys_B_ intramolecular disulfide first because only the GSSG treatment led to the formation of a glutathionylated protein and to fluorescence quenching and second because the WT protein was crystallized with a glutathionylated adduct. However, at that time, we did use neither H_2_O_2_ and GSNO treatments, nor prolonged incubation, nor alkylation assays. In fact, reconsidering the results of mass spectrometry showing, in addition to the peaks with one or two glutathione adducts, a peak with a decrease of 2 Da between the reduced protein and the GSSG-treated protein, it is very likely that a small fraction of the protein contained an intramolecular disulfide formed between active site cysteines (Couturier et al., 2011). Here, we have clearly observed that a prolonged oxidative treatment of reduced class I Grxs with H_2_O_2_, GSSG, or GSNO led preferentially to the formation of an intramolecular disulfide bond between Cys_A_ and Cys_B_. For the H_2_O_2_ treatment, it likely goes first through the formation of a sulfenic acid on Cys_A_ before Cys_B_ performs a nucleophilic attack on it. For the GSSG or GSNO treatments, it is very likely that a nucleophilic attack of Cys_A_ onto these oxidized glutathione species leads to the formation of a glutathione adduct on Cys_A_ which is then slowly reduced by a Cys_B_ nucleophilic attack leading to the formation of the intramolecular disulfide and to the concomitant release of a GSH molecule.

It is worth mentioning here that these *in vitro* experiments have been done in particular with the aim of exploring all possible post-translational modifications but the conditions are not exactly physiological first because the concentrations of the oxidants may not be realistic and more importantly because this is done in the absence of GSH, which is normally found at high amounts and is highly reduced in most cell compartments. The presence of GSH would certainly prevent to a large extent the formation of these intramolecular disulfides because it may preferentially reduce either the sulfenic acids or the glutathione adducts over the Cys_B_ recycling cysteine. This is certainly the case for GrxC3 and GrxC4 since mutating CysB has no effect on the activity. The formation of an intramolecular disulfide in these proteins could thus constitute uniquely a side reaction, protecting the cysteine or inactivating the proteins only under specific conditions. On the contrary, if we consider that the increase of protein activity observed for CysB mutated GrxC1 and GrxC2 in HED and DHA assays in the presence of a fully reduced GSH pool reflects the formation of this intramolecular disulfide, it may be physiologically relevant for GrxC1 and GrxC2. This is interesting in several respects. First, while the current dogma is that the preferential substrates of Grxs are glutathionylated proteins, recent studies performed with mammalian Grx2 indicated that they can reduce dithiol substrates, e.g., proteins with intra- or intermolecular disulfide (Hanschmann et al., 2010; Schutte et al., 2013). In this case, the Grxs should employ a reaction mechanism similar to the one used by Trxs involving both Cys_A_ and Cys_B_. Second, based on the observation that some Grxs can be reduced by thioredoxin reductases (Johansson et al., 2004; Zaffagnini et al., 2008; Couturier et al., 2013), another possibility could be that in specific physiological situations or sub-cellular compartments where glutathione is depleted, absent or oxidized, the formation of an intramolecular disulfide may be determinant either to favor the use of an alternative reducing system as the one constituted by NADPH and thioredoxin reductase or to simply constitute a protective mechanism, preventing the irreversible overoxidation of this residue and thus protein inactivation.

In conclusion, the role of Cys_B_ in dithiol Grxs is yet uncertain and may depend on the Grx isoform as this residue influences differentially the protein activity. The fact that the formation of an intramolecular disulfide bond between Cys_A_ and Cys_B_ is possible in all cases lets open the possibility to use both monothiol and dithiol catalytic mechanisms, contrary to Grxs possessing only one cysteine residue.

### The role of the semi-conserved Cys_*C*_ in GrxC1 and GrxC2 isoforms

The Cys_*C*_ cysteine residue, present in the IGGCD motif (at position 88 and 80 in GrxC1 and C2, respectively), is found in several other class I Grxs, including plant GrxC5 and S12 as well as in human Grx1 and *E. coli* Grx3. Moreover, it is also present in many class II Grxs. It was shown for instance that a Cys_A_-Cys_*C*_ intramolecular disulfide bond can be formed in *S. cerevisiae* Grx5 and *C. reinhardtii* Grx3 (Tamarit et al., 2003; Zaffagnini et al., 2008), suggesting that Cys_*C*_ can serve as a recycling cysteine in the absence of Cys_B_. Reduced glutathione does not seem to be an efficient reductant for this intramolecular disulfide bond and in the case of CrGrx3, it is reduced by a ferredoxin-thioredoxin reductase system (Zaffagnini et al., 2008). Although GrxS12 has a monothiol WCSYS active site sequence, a Cys_A_-Cys_*C*_ intramolecular disulfide has not been observed whatever the oxidizing conditions tested. Moreover, in the crystal structure of a glutathionylated GrxS12, the sulfur atom of Cys87 is 9.6Å away from the Cys27-SG adduct (Couturier et al., 2009b). Hence, this cysteine may not serve as a recycling cysteine in class I Grxs and does not *a priori* participate to the reaction mechanism. Consistently, its mutation in GrxC1 and C2 did not influence protein activity, as previously observed for poplar GrxS12, Arabidopsis GrxC5, or *E. coli* Grx3 (Nordstrand et al., 1999; Couturier et al., 2009b, 2011).

However, considering its position very close to the active site residues and to residues involved in GSH binding an effect on activity could have been observed. Indeed, it is positioned between the so-called GG kink which is important in determining the backbone geometry of the following amino acids, and a Thr or an Asp which forms hydrogen bonds with GSH (see Figure 1) (Couturier et al., 2009b; Li et al., 2010). In the GrxS12 structure, the backbone amino group of Cys_*C*_ also directly forms a hydrogen bond with the glutamyl group of GSH (Couturier et al., 2009b), but the substitution of the cysteine into serine would not disrupt this interaction, preventing to definitely rule out a GSH stabilizing effect of this cysteine. On the other hand, it is probable that modifying the redox state of this cysteine might have more pronounced effects influencing for example GSH binding. It has been shown for example that, under non-reducing conditions, *E. coli* Grx3 exists under an expected monomeric form but also forms disulfide-bridged dimer or multimers via Cys_*C*_ (Aslund et al., 1994, 1997). In addition, previously reported oxidative treatments of human Grx1, which contains three extra active site cysteines including Cys_*C*_, led to the identification of several possible post-translational modifications, i.e., intramolecular disulfide, disulfide-bonded dimers and oligomers, glutathione adducts, nitrosylation, some of them inhibiting its activity (Hashemy et al., 2007). Depending on the conditions, Cys_*C*_ was found to be either nitrosylated or glutathionylated or involved into a disulfide. Similarly, we have observed redox changes of GrxC1 and C2 upon treatment with oxidants that could further provide clues about the possible function of this cysteine, if any. In the presence of H_2_O_2_, this cysteine was found to be either overoxidized or involved in disulfide-bridged homodimers. Since this dimerization only occurs in presence of H_2_O_2_, the formation of an intermediate sulfenic acid is likely required before intermolecular disulfide bond formation which is consistent with the presence of a small part of overoxidized proteins. Interestingly, GSH can efficiently reduce these disulfide-bridged dimers, making this reaction physiologically reversible. In the presence of GSSG or GSNO, Cys_*C*_ is also prone to oxidative modification, but in the form of a glutathione adduct. Although we cannot test the impact of these modifications on Grx activity (we cannot selectively modify Cys_*C*_ without also oxidizing Cys_A_and Cys_B_), we can speculate that the different reversible oxidation forms of Cys_*C*_ observed here might represent regulatory or signaling intermediates transiently modifying the functions of these Grxs by affecting for example the nature or the time-course reduction of the target proteins.

### Conflict of interest statement

The authors declare that the research was conducted in the absence of any commercial or financial relationships that could be construed as a potential conflict of interest.

## Supplementary material

Figure S1 **Electrospray ionization mass spectrometry analysis of PtGrxC1 WT**. Spectra of whole protein were determined for reduced protein before **(A)** and after treatment with GSSG **(B)**, GSNO **(C)**, or H_2_O_2_ **(D)** as described in the Methods section.

Figure S2 **Electrospray ionization mass spectrometry analysis of PtGrxC2 WT**. Spectra of whole protein were determined for reduced protein before **(A)** and after treatment with GSSG **(B)**, GSNO **(C)**, or H_2_O_2_ **(D)** as described in the Methods section.

Figure S3 **Electrospray ionization mass spectrometry analysis of PtGrxC3 WT**. Spectra of whole protein were determined for reduced protein before **(A)** and after treatment with GSSG **(B)**, GSNO **(C)**, or H_2_O_2_ **(D)** as described in the Methods section.

Figure S4 **Electrospray ionization mass spectrometry analysis of PtGrxC4 WT**. Spectra of whole protein were determined for reduced protein before **(A)** and after treatment with GSSG **(B)**, GSNO **(C)**, or H_2_O_2_ **(D)** as described in the Methods section.

Figure S5 **Electrospray ionization mass spectrometry analysis of PtGrxC1 C88S**. Spectra of whole protein were determined for reduced protein before **(A)** and after treatment with GSSG **(B)**, GSNO **(C)**, or H_2_O_2_ **(D)** as described in the Methods section.

Figure S6 **Electrospray ionization mass spectrometry analysis of PtGrxC2 C80S**. Spectra of whole protein were determined for reduced protein before **(A)** and after treatment with GSSG **(B)**, GSNO **(C)**, or H_2_O_2_ **(D)** as described in the Methods section.

Table S1 **Primers used for site-directed mutagenesis**.
