# Supplementary figures and images for: Toward a refined classification of class I dithiol glutaredoxins from poplar: biochemical basis for the definition of two subclasses
Source: Front Plant Sci. 2013 Dec 18;4:518. doi: 10.3389/fpls.2013.00518 (PMC3866529; doi:10.3389/fpls.2013.00518)

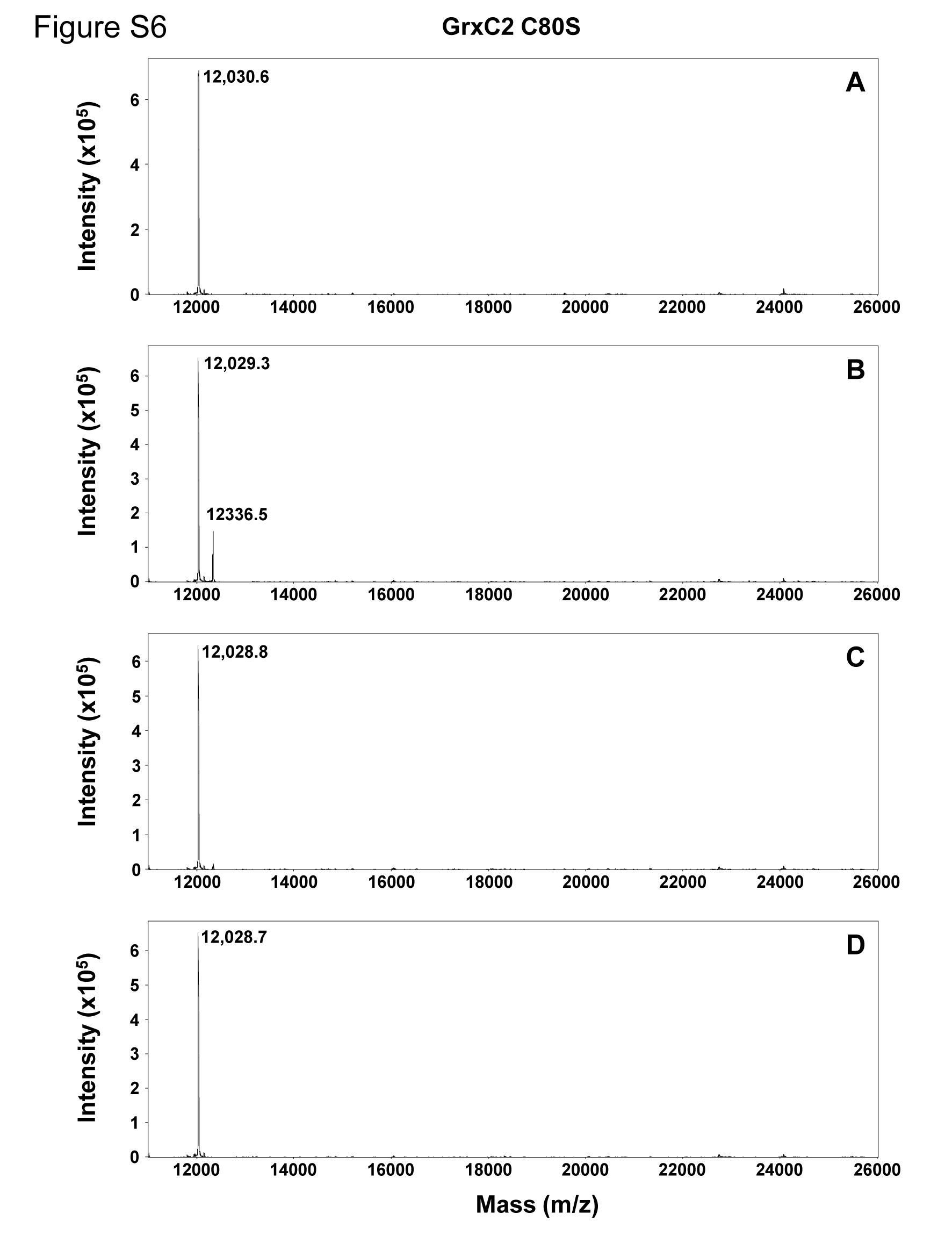

Supplement: Figure S1 — Electrospray ionization mass spectrometry analysis of PtGrxC1 WT. Spectra of whole protein were determined for reduced protein before (A) and after treatment with GSSG (B), GSNO (C), or H2O2 (D) as described in the Methods section. [file DataSheet1.ZIP › S6.TIF]

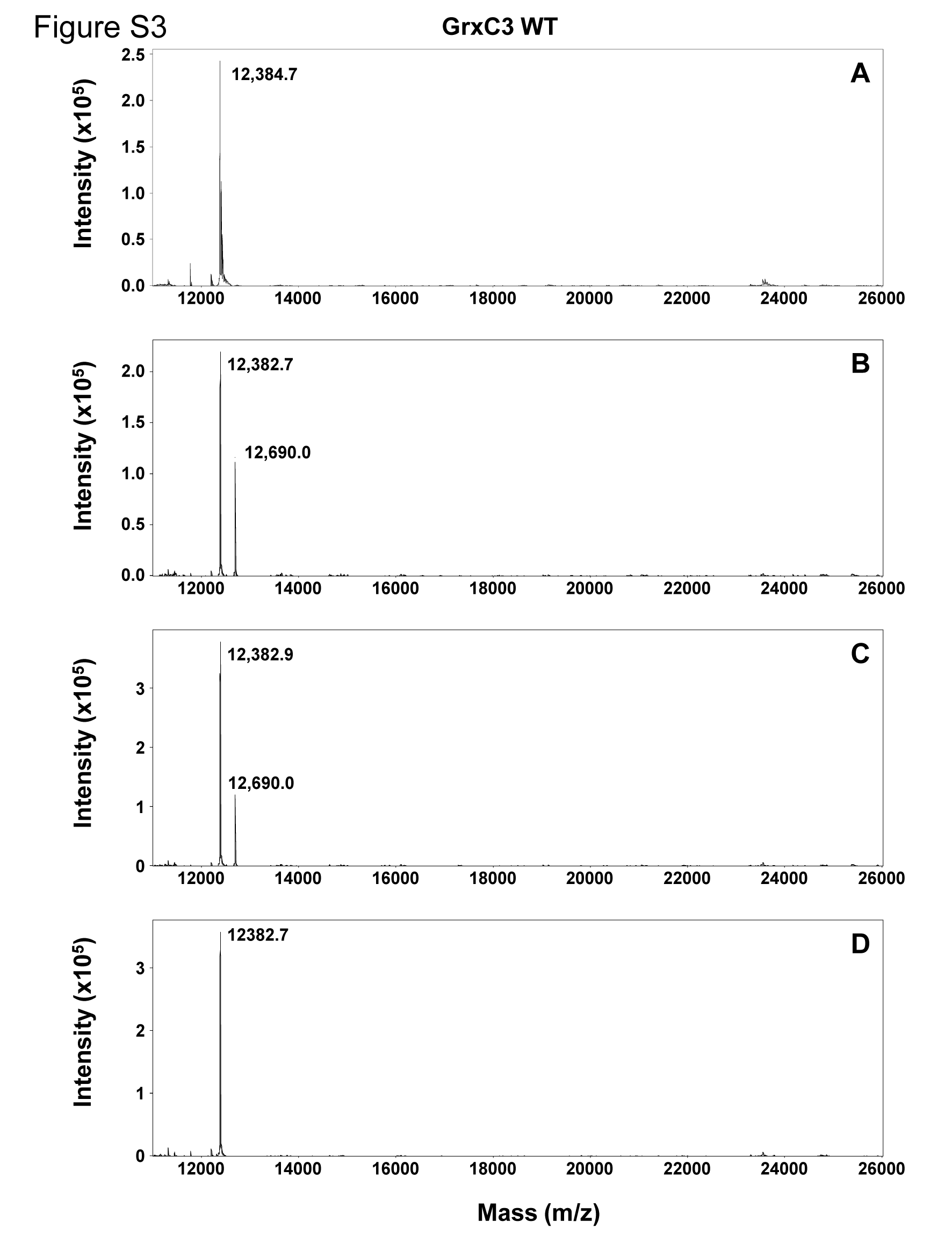

Supplement: Figure S1 — Electrospray ionization mass spectrometry analysis of PtGrxC1 WT. Spectra of whole protein were determined for reduced protein before (A) and after treatment with GSSG (B), GSNO (C), or H2O2 (D) as described in the Methods section. [file DataSheet1.ZIP › S3.TIF]

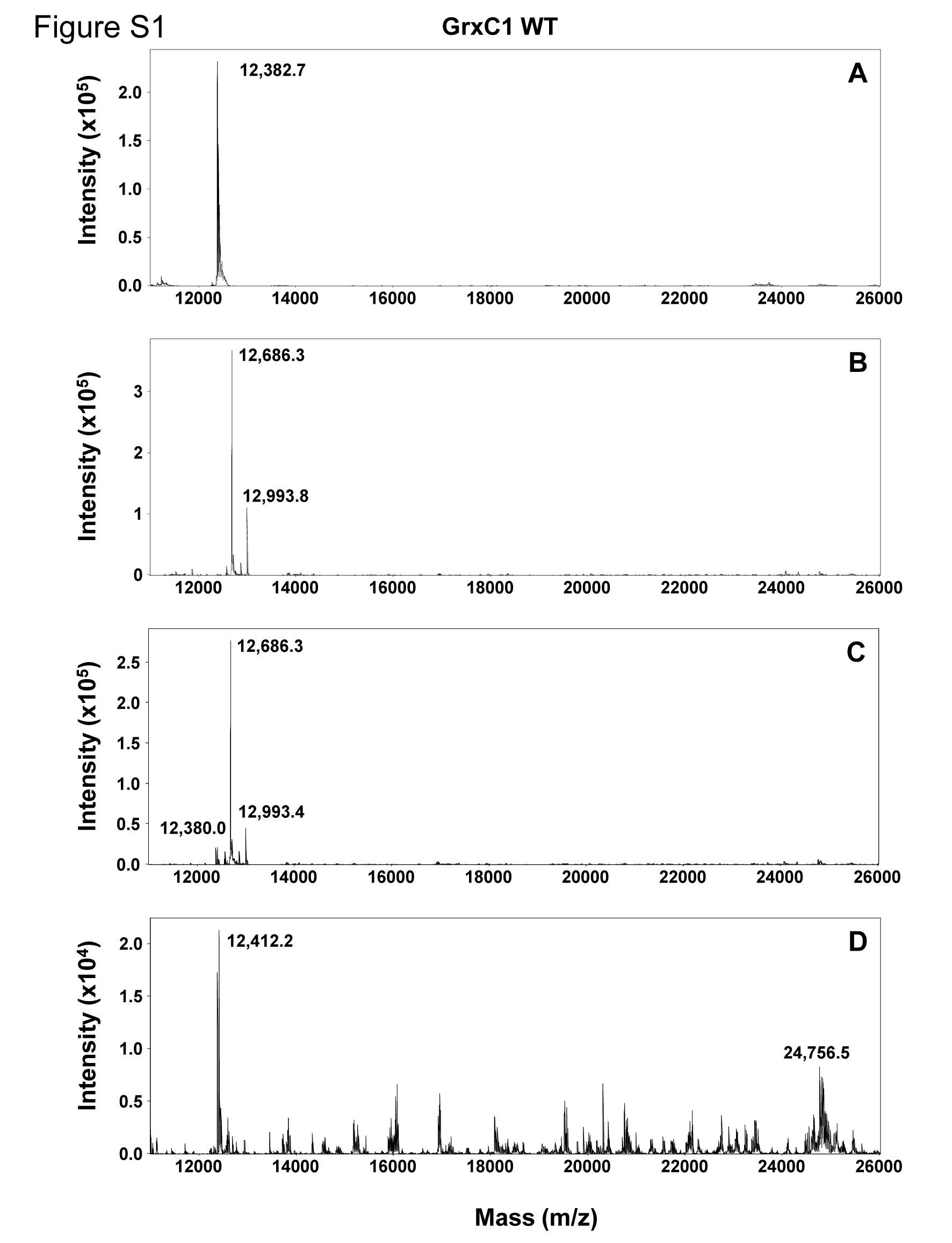

Supplement: Figure S1 — Electrospray ionization mass spectrometry analysis of PtGrxC1 WT. Spectra of whole protein were determined for reduced protein before (A) and after treatment with GSSG (B), GSNO (C), or H2O2 (D) as described in the Methods section. [file DataSheet1.ZIP › S1.TIF]

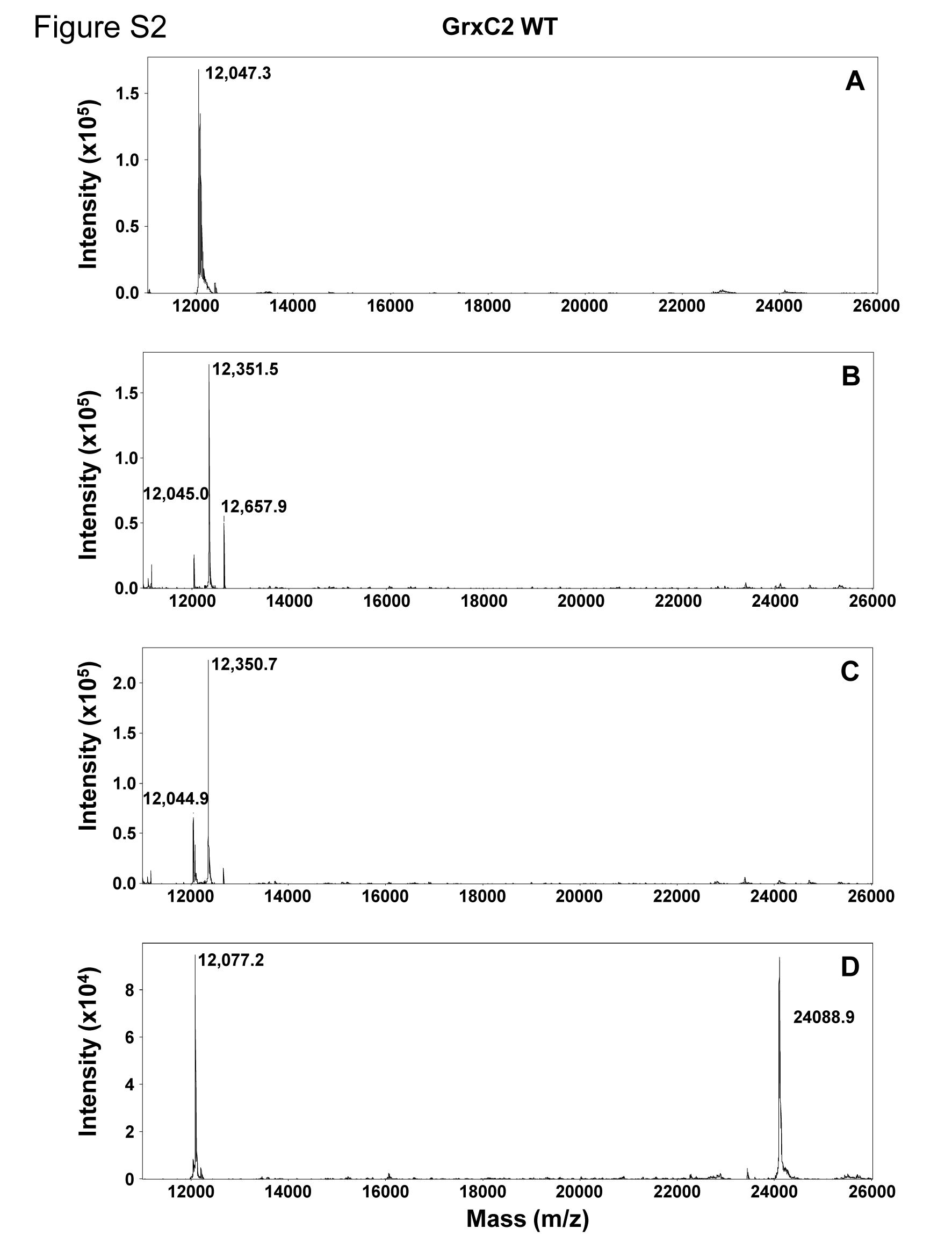

Supplement: Figure S1 — Electrospray ionization mass spectrometry analysis of PtGrxC1 WT. Spectra of whole protein were determined for reduced protein before (A) and after treatment with GSSG (B), GSNO (C), or H2O2 (D) as described in the Methods section. [file DataSheet1.ZIP › S2.TIF]

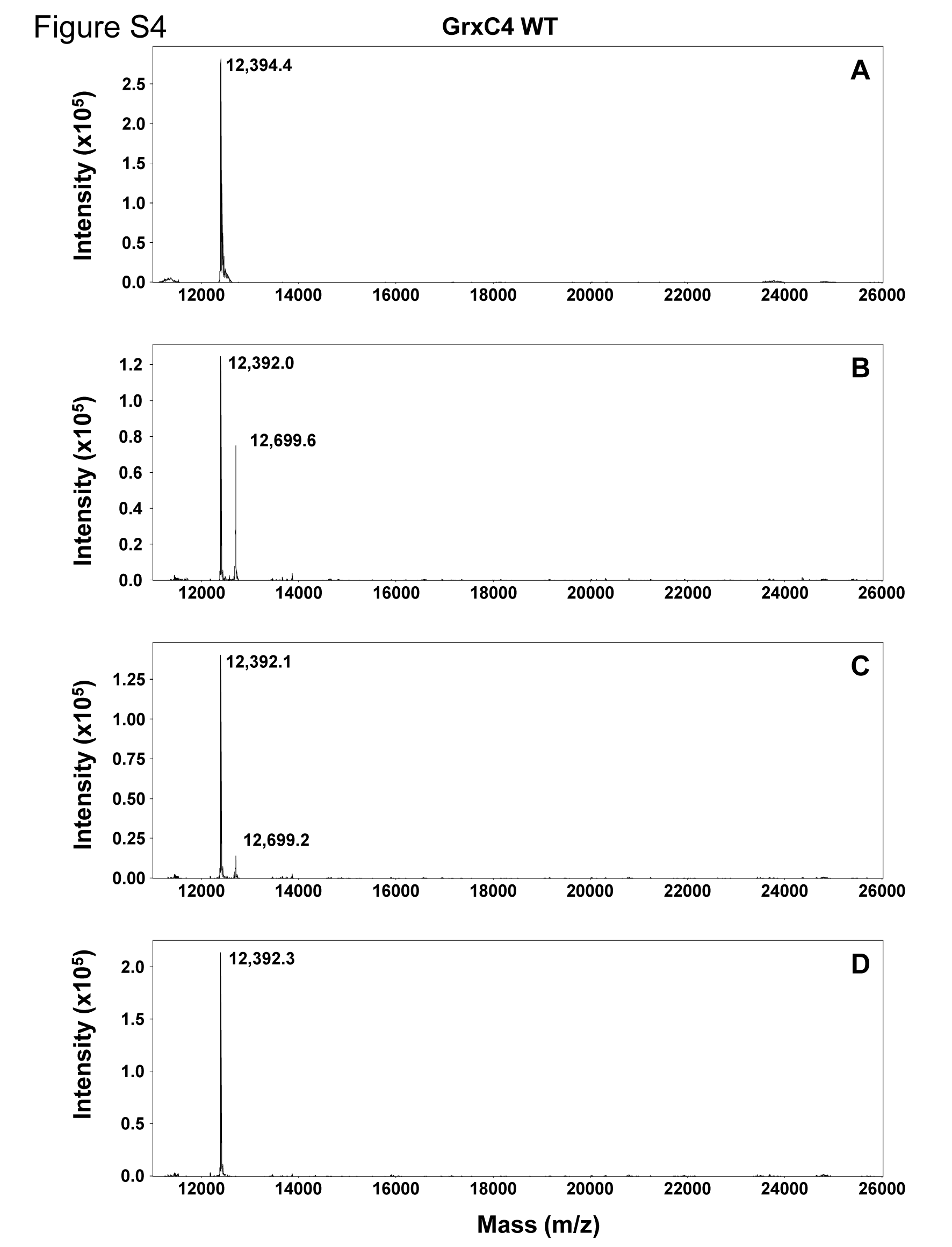

Supplement: Figure S1 — Electrospray ionization mass spectrometry analysis of PtGrxC1 WT. Spectra of whole protein were determined for reduced protein before (A) and after treatment with GSSG (B), GSNO (C), or H2O2 (D) as described in the Methods section. [file DataSheet1.ZIP › S4.TIF]

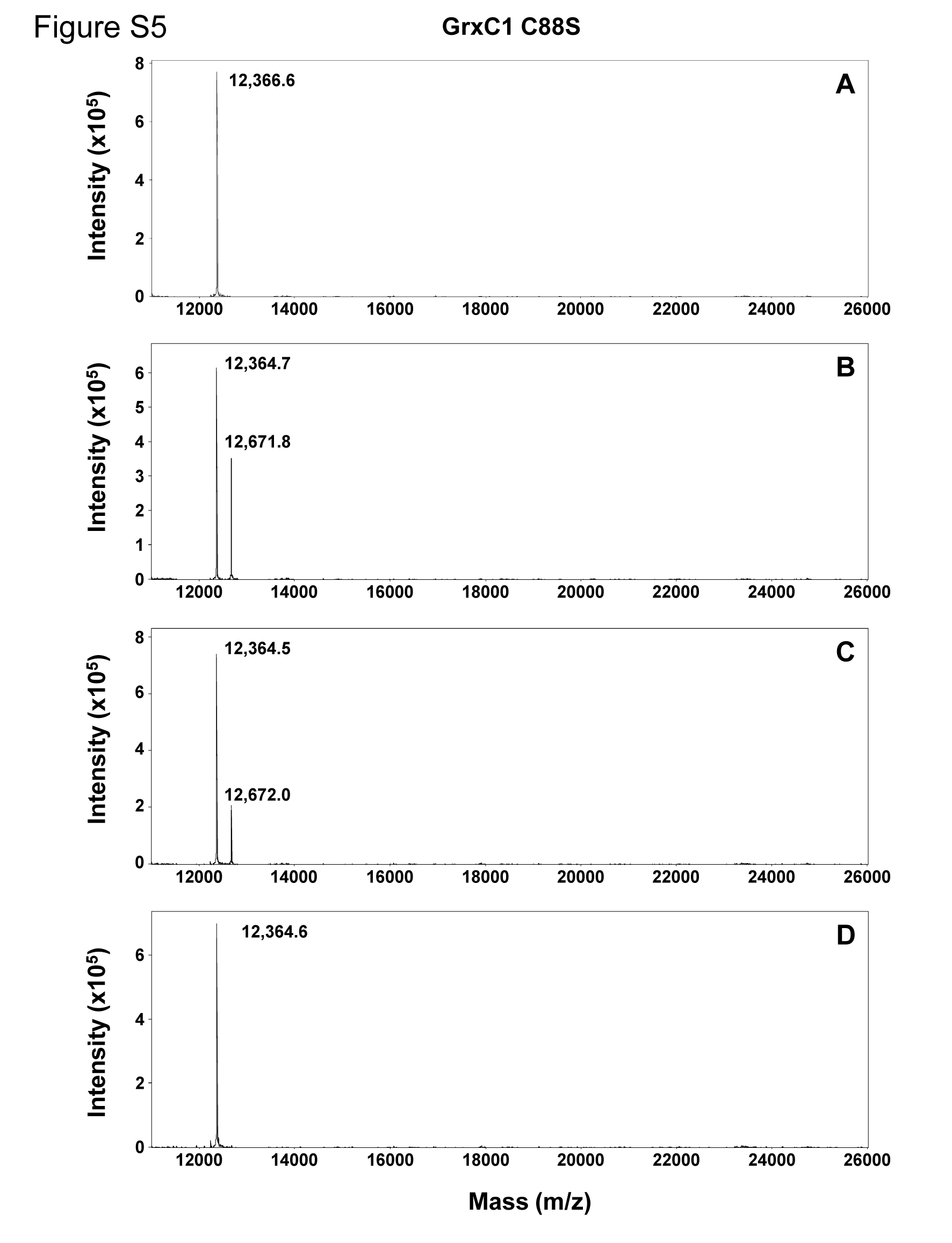

Supplement: Figure S1 — Electrospray ionization mass spectrometry analysis of PtGrxC1 WT. Spectra of whole protein were determined for reduced protein before (A) and after treatment with GSSG (B), GSNO (C), or H2O2 (D) as described in the Methods section. [file DataSheet1.ZIP › S5.TIF]
